# The effectiveness in preventing frailty of exercise intervention provided by community pharmacists to older persons with chronic conditions: A pragmatic randomized controlled trial

**DOI:** 10.1186/s12877-023-03858-6

**Published:** 2023-04-07

**Authors:** Noritake Hirota, Hiroshi Okada, Noboru Okamura

**Affiliations:** 1 Department of Clinical Pharmacy, School of Pharmacy and Pharmaceutical Sciences, Mukogawa Women’s University, Nishinomiya, Japan; 2 Aozora Pharmacy, General Incorporated Association Osaka Pharma Plan, Osaka, Japan; 3 Department of Health Informatics, School of Public Health, Kyoto University, Kyoto, Japan

**Keywords:** Community Pharmacy, Frailty prevention, Pragmatic RCT

## Abstract

**Background:**

Once older persons become frail, the risk of falls, bone fractures, and other problems increases. Exercise intervention is a form of prevention that has a high degree of evidence.

**Objective:**

We investigated the effectiveness of frailty prevention consisting of exercise intervention by community pharmacists at 11 pharmacies operated by Osaka Pharma Plan.

**Methods:**

In total, 103 older persons between 70 and 79 years of age (53 males and 50 females) who were suffering from chronic conditions and who visited one of 11 pharmacies between January and March 2021 were enrolled. They were then randomly assigned to either the Intervention group (IG: 6 pharmacies, 61 patients) who were subjected to intervention by a pharmacist, or the Usual Care group (UG: 5 pharmacies, 42 patients) who were not subjected to intervention. At the beginning of the trial and 6 month after, their muscle mass, etc. were measured using a body composition meter, and their Five-Times Sit-To-Stand Test results were also measured. Patients in the IG were provided with information by way of leaflets during the time they were guided regarding taking their medication for a period of one to six months that encouraged exercising at home. Those in the UG were given the standard guidance related to taking their medication.

**Results:**

The amount of change in muscle mass was 1.08 ± 7.83% (95%CI: -1.24-3.41) in IG and − 0.43 ± 2.73% (95%CI:-1.58-0.72) in UG, indicating that there was a trend toward an increase in IG. The percent change in the Five Times Sit-To-Stand Test times at + 6 M was − 0.002 ± 0.24% (95%CI: -0.09-0.05) in IG and − 0.04 ± 0.21% (95%CI:-0.13-0.07) in UG, but in cases in which the second measured time was faster than the first measured time, the results were 65.2% for IG and 29.2% for UG, indicating a significant difference (p = 0.00563).

**Conclusion:**

Despite the fact that the amount of time community pharmacists can devote to providing guidance on taking medications is limited, it has been previously reported that providing information to patients causes a change in patient behavior. The results of the present study are highly significant as they suggest the possibility that this may hold true even when used to prevent frailty, based on the evidence obtained.

**Trial registration:**

This trial was registered at UMIN-CRT on 1st of January, 2021. The registration number is UMIN000042571.

## Background

As the global population rises, the percentage of those aged 65 years and older (the aging rate) has increased markedly. The global aging rate was 5.1% in 1950 but is expected to increase to 17.8% by 2060 [1]. The Japanese aging rate was low among the advanced nations of the world in the 1980s and was around average in the 1990s, but by 2005 it had reached its highest point ever [1]. In response to the advancing aging rate in Japan, the Science Council of Japan announced its recommendations from a scientific perspective in 2020. These recommendations emphasized the promotion of anti-frailty measures for the elderly from both a medical and a community-building perspective [2]. In turn, the Ministry of Health, Labour and Welfare of Japan created comprehensive community care systems based on middle school districts. These systems would help create environments in which the elderly can be assured of medical care and nursing care in their familiar communities [3].

The definition and concept of “frailty” differ from country to country, but in Japan, the term indicates “a state in which a person experiences a decline in the ability to recover from stress due to age-associated declines in reserve capacity” [4]. In Europe and the United States, it is defined as “a state of weakness in which one experiences a decline in the ability to recover homeostasis after experiencing stress” and “a state in which there is a higher risk of health disorders, including falls, physical disabilities, and death” [5, 6]. And international definitions of frailty is as “a medical syndrome with multiple causes and contributors that is characterized by diminished strength, endurance, and reduced physiologic function that increases an individual’s vulnerability for developing increased dependency and/or death.”[7].

What they have in common is the fact that frailty is described as a state somewhere between healthy and in need of nursing care. According to a systematic review conducted outside Japan, the frailty rate among community-dwelling people aged 65 years and older is 9.9%, this percentage increases with increased age, and more women than men are prone to frailty [8]. In Japan, the frailty rates by age are 4.0% for those aged 65 to 74, 16.2% for those aged 75 to 84, and 34.0% for those aged 85 years and older, and it has been reported that the frailty rate among older persons who visit the hospital on an outpatient basis for chronic conditions is 21.6% [9]. If left untreated, frailty is known to lead to increases in the risk of falls, bone fractures, postoperative complications, and the necessity for nursing care, dementia, institutionalization, and death [10]. Exercise intervention is recommended as a way to prevent the onset of frailty that has a high degree of evidence [11].

In terms of what community pharmacies can do in the effort to create comprehensive community care systems, based on the “Pharmacy Vision for Patients” produced by the Ministry of Health, Labour and Welfare of Japan in October 2015 [12], a system of health support pharmacies was begun in October 2016 and then in August 2021 a system of community collaboration pharmacies was created. In particular, with regard to pharmacies that function as health support pharmacies, community pharmacists provide health promotion services to their communities while simultaneously playing an important role in providing frailty prevention services to the elderly. There has already been a study on measures implemented by community pharmacies to deal with frailty using nutrition assessments of community residents aged 65 years and older [13] as well as a study on pharmacist intervention in oral frailty and the ability to swallow medication [14]. However, there have been nearly no randomized controlled trials (RCT) on exercise intervention by pharmacists at community pharmacies.

Thus, the present study was an investigation of whether or not the muscle mass of patients increased as a result of the promotion of daily exercise by providing them with simple information on frailty prevention measures every time they received guidance on taking medication from a community pharmacist. The patients were aged between 70 and 79 years who suffered from chronic conditions and who utilized the services of 11 community pharmacies operated by the General Incorporated Association Osaka Pharma Plan. The study investigated whether it was possible for community pharmacies to provide support for the purpose of maintaining the functions of older persons.

## Methods

### Study design

This study was a randomized controlled trial without masking. Patients were masked, pharmacists were not. The reason is that pharmacists are employees of the same pharmacy group. However, pharmacists in the control and intervention groups did not exchange information during the trial. The randomized controlled trial (RCT) was implemented at community pharmacies operated by Osaka Pharma Plan. The pharmacies were located in the cities of Osaka and Suita, and as all the pharmacies were in urban areas, there were no regional differences between them. In this study, patients were randomized to the control or intervention group. The two groups were the Intervention group (IG), which was provided with intervention (information) about exercise by pharmacists, and the Usual Care group (UG: control group), which received only routine medication guidance. The 11 pharmacies that participated in this study were randomly assigned to either the IG or the UG after patients meeting the conditions indicated below were enrolled. In addition, patients enrolled in this study were not previously assessed for frailty.

### Patient enrollment conditions and enrollment target

#### Enrolled patients

Patients from among those who visited the community pharmacies participating in this study every month for the purpose of having prescriptions filled were enrolled in this study after meeting the following conditions: They were aged between 70 and 79 years; they were prescribed medications for chronic conditions such as hypertension, diabetes, and hyperlipidemia; and they provided their consent to participate in this study.

#### Exclusion criteria

The following patients were excluded from enrollment in this study: (1) those receiving treatment for cancer that may lead to malnutrition and a higher fracture risk, (2) Patients taking osteoporosis medication, 3.Patients prescribed antidepressants and antipsychotics, 4. Patients taking steroids, 5. Patients prescribed dementia medication, 6. Obese patients with a BMI of 30% or higher, and 7. Patients advised by their attending physician to limit exercise.

#### Sample size and randomization

Regarding increases in muscle mass, which is the primary endpoint, the sample size was calculated using EZR ver. 1.27 [15] with statistically significant differences between the IG and the UG of under 5% (p < 0.05) and a detection rate of 80% with 46 patients per group. We assumed a dropout rate during the trial of approximately 20%, and thus to achieve 50 patients per group, our target enrollment was 60 patients per group (a total of 120 patients). At the time of enrollment, the male-to-female ratio was 1:1.

Simple randomization was used for assigning the subjects to the IG and UG groups. Randomization and allocation will be performed by researchers not involved in data collection. Research assistants involved in data collection and processing will be blinded for group allocation.

#### Education/Training of pharmacists

Prior to the start of the trial, pharmacists working at IG pharmacies viewed a 30-minute video created by the joint researcher HO entitled “Nudging techniques for guiding patients toward exercising for the purpose of preventing frailty.” The contents of the video included basic nudging techniques and methods of communicating that do not force guidance upon patients when the pharmacist provides guidance on taking medication.

**Fig. 1:**
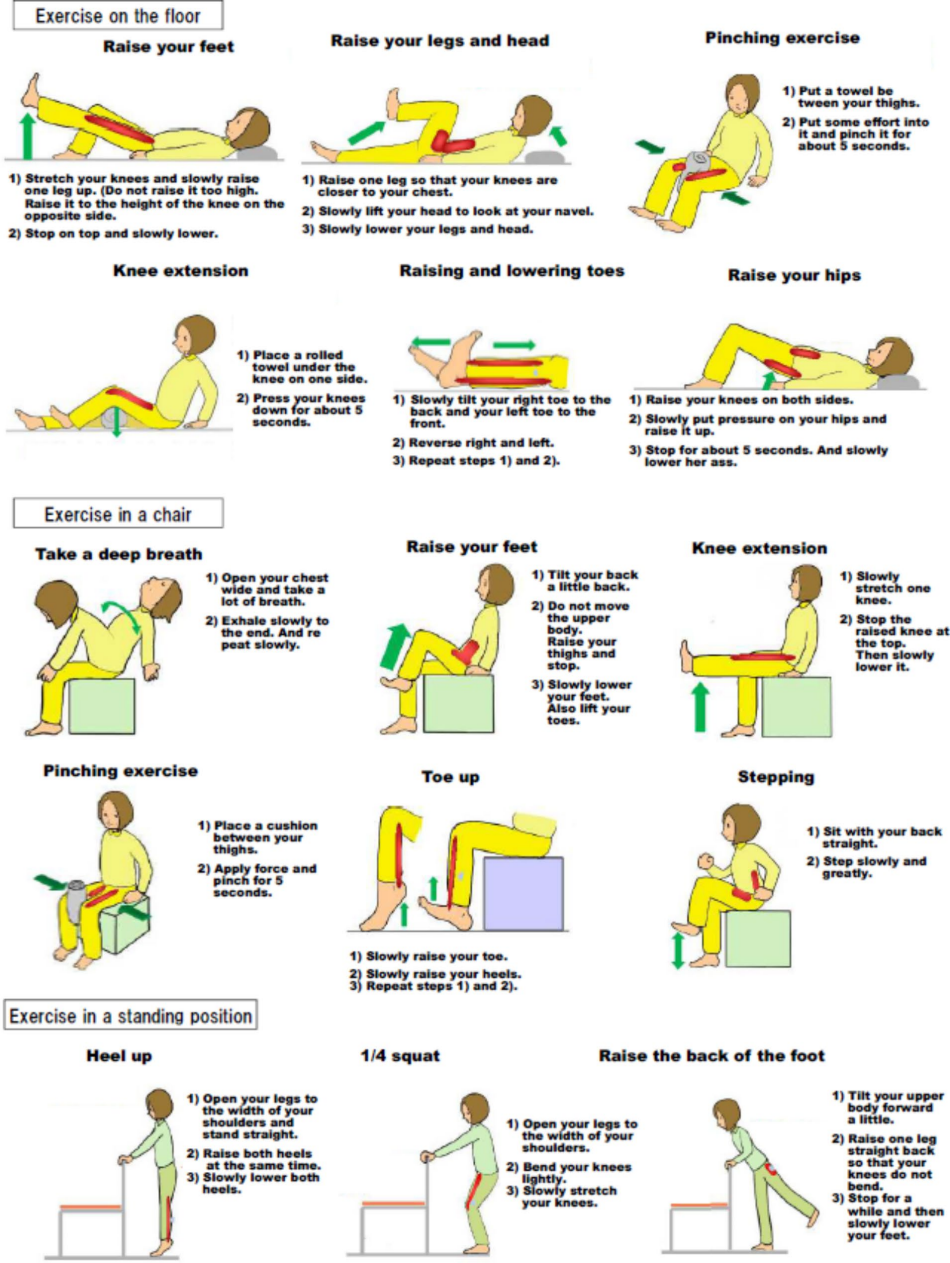
we distributed exercise documentation to patients

The term “nudge” means “to gently prod someone to pay attention to something.” The nudge theory was advocated in 2008 by US economist Professor Richard Thaler and legal scholar Professor Cass Sunstein　[16]. It has since been utilized to change consumers’ purchasing behaviors. Therefore, in this study, at the IG pharmacy, the pharmacist handed out the leaflets (Fig. 1) to the patients each time they instructed them to take their medications and encouraged them to exercise at home. The supervising pharmacist never specified the type of exercise; rather, the patients chose their preferred exercises.

#### Contents of pharmacist intervention

NH created the materials for at-home exercises that were distributed to patients based on “Let’s start! Let’s move!” (Fig. 1) [17]. The materials contained descriptions of how to do the following exercises: “Floor Exercises” (6 types: foot lifting, foot and head lifting, “scissors exercise,” knee extension, toe raising and lowering exercise, butt raising exercise), “Chair Exercises” (6 types: deep breathing, foot lifting, knee extension, “scissors exercise,” toe raising exercise, stepping exercise), and “Standing Exercises” (3 types: heel lift, one-quarter squats, raising the foot toward the back).

In the case of the IG pharmacies, the pharmacist provided an explanation of the contents of the exercises during the patient’s first visit. When providing the patients with guidance for taking their medication on their second and subsequent visits, the pharmacists asked whether the patients were able to do the exercises at home. During each patient visit, the pharmacists distributed the same material and encouraged the patients to exercise. However, the pharmacists did not force a specific exercise program on the patients. At the UG pharmacies, the pharmacists provided routine guidance for taking medication; however, they did not distribute the exercise materials.

#### Assessments of health status, muscle mass, and frailty

**Fig. 2:**
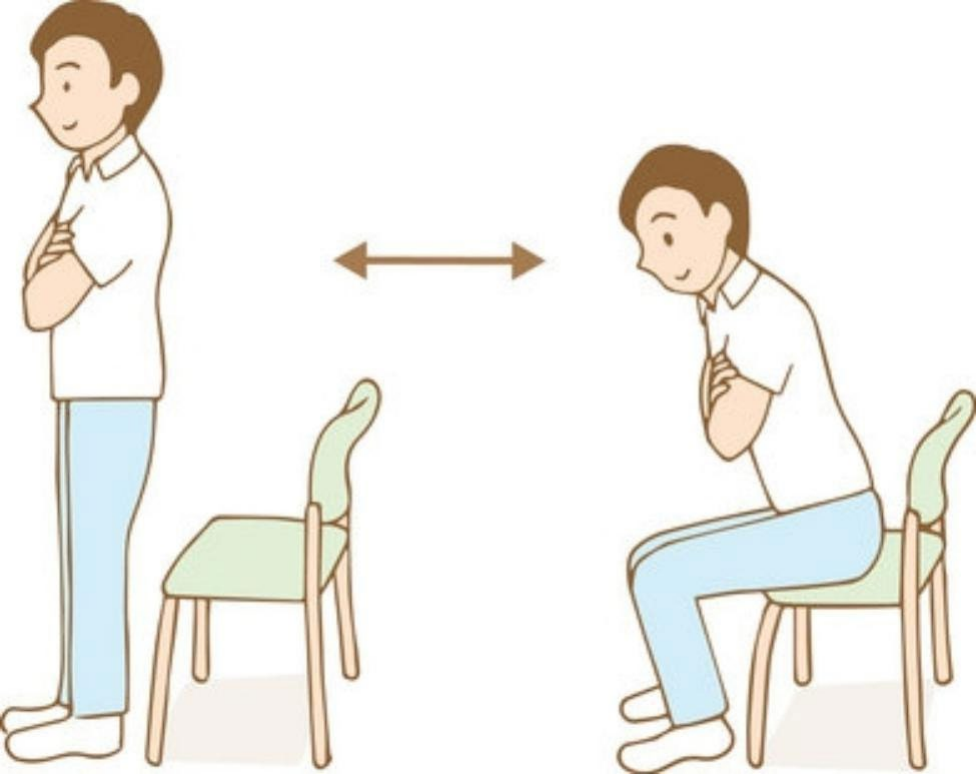
Schematic of the Five-Times Sit-To-Stand Test

At the time the patients were enrolled, we asked them to complete the “Health Status Questionnaire” created by the Ministry of Health, Labour and Welfare of Japan [18]. We also measured the patients’ height, and, using a body composition meter (Tanita RD-800), we measured muscle mass (total of the trunk, right arm, left arm, right leg, and left leg), body weight, body fat percentage, BMI, and estimated bone mass. We also measured the Five Times Sit-To-Stand Test time results. During their visit six months later (+ 6 M), we once again measured their body composition and their Five-Times Sit-To-Stand Test timings. The Five-Times Sit-To-Stand Test is a test that measures the muscle strength of the lower extremities by measuring the time it takes to stand up from a chair five times. Stand up and sit down with maximum effort five times and measure the time (in seconds) it takes. Start the measurement “when the subject’s body starts to move” and end the measurement “when the subject stands upright for the fifth time” (Fig. 2).

#### Assessment methods

The primary endpoint was assessed using the percent (%) change in muscle mass obtained from body composition measurements taken at + 6 M. The secondary endpoints were assessed as follows: We measured the percent (%) change at + 6 M in the Five Times Sit-To-Stand Test time results (secs) and the percent (%) change at + 6 M in the body weight, body fat percentage, BMI, and estimated bone mass as measured using body composition meter.

#### Analytical methods

The percent (%) changes in all measured values from the initial measurement to + 6 M were tested for statistically significant differences using the Mann-Whitney U test. At the same time, we compared the number of patients whose muscle mass showed increases at + 6 M to the percentage of patients whose Five Times Sit-To-Stand Test times (secs) decreased at + 6 M using the chi-squared test. Statistical processing was performed using EZR ver. 1.27 [15]. The significance level was set so that a significant difference was under 5% (p < 0.05).

## Results

### The number of enrolled patients and the number of patients who underwent muscle mass measurements at + 6 M

**Fig. 3:**
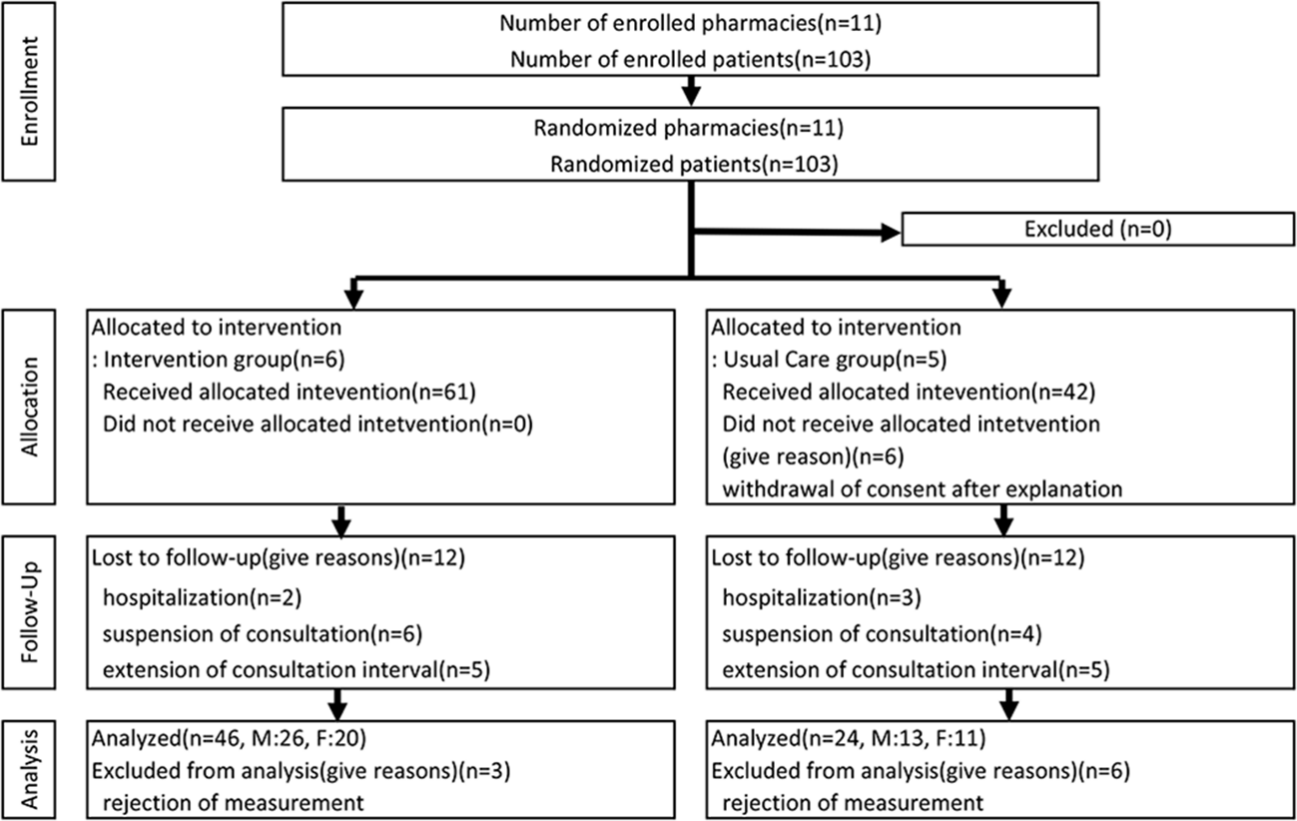
Number of enrolled patients in each group and number of patients whose muscles mass could be measured after 6 months

In total, 103 patients (53 males, 50 females) at 11 pharmacies were enrolled in this study after granting their consent. The 11 pharmacies were randomly assigned into two groups, with the IG consisting of 6 pharmacies and 61 patients (32 males, 29 females) and the UG consisting of five pharmacies and 42 patients (21 males, 21 females). The final number of patients in each group whose muscle mass was measured at + 6 M was 46 in IG (26 males, 20 females) and 24 in UG (13 males, 11 females; Fig. 3). Dropouts during the study totaled 15 patients in IG (6 males, 9 females) and 18 in UG (9 males, 9 females). The reasons for dropping out were nearly the same in both groups and included longer gaps between examinations, hospitalization, and refusal to be measured (Fig. 3).

### Comparison of patient backgrounds in the IG and UG at enrollment

No significant differences were found between IG and UG in terms of the following enrolled patient background characteristics: Age, height, weight, muscle mass, body fat percentage, BMI, estimated bone mass, and body water percentage (Table 1).

**Table 1:** Comparison of patient background at enrollment in the intervention group and the usual care group

items	sex	Intervention Group	Usual Care Group
Age	M	76.0 ± 2.8	74.5 ± 3.0
	F	76.5 ± 1.5	75.2 ± 2.5
Height (cm)	M	16.2 ± 3.9	16.5 ± 5.8
	F	15.2 ± 5.5	153.4 ± 3.8
Body weight(Kg)	M	62.4 ± 6.0	63.8 ± 7.8
	F	54.7 ± 9.0	53.9 ± 8.4
Muscle mass(Kg)	M	45.5 ± 4.1	36.5 ± 7.1
	F	36.5 ± 7.1	34.4 ± 3.0
Body fat percentage(%)	M	25.8 ± 7.8	23.5 ± 7.0
	F	33.0 ± 7.1	30.9 ± 7.8
BMI(%)	M	23.7 ± 1.9	23.5 ± 7.0
	F	23.4 ± 3.2	22.7 ± 3.2
Estimated bone mass(Kg)	M	2.5 ± 0.2	2.5 ± 0.3
	F	2.0 ± 0.3	2.0 ± 0.3
Body water content(%)	M	47.8 ± 6.1	46.9 ± 4.3
	F	46.9 ± 4.3	48.1 ± 5.9

The health status questionnaires were scored and IG and UG were compared. This comparison indicated that there were no significant differences between the two groups for any item (Table 2). Based on these findings, we determined that the patient background characteristics in both groups at the start of the study were the same.

**Table 2:** Comparison of health status at enrollment between the intervention group and the usual care group

Questions				Score (Mean ± SD)	
				Intervention Group	Usual Care Group
	1. How is your current health?	Good : 5, Fairly good : 4, Normal : 3, Not very good : 2, Not good : 1		3.50 ± 1.05	5.50 ± 0.98
	2. Are you happy with your daily life?	Satisfaction:5, Slightly satisfied:4, Slightly dissatisfied:3, Dissatisfied: 2		4.31 ± 0.79	4.04 ± 0.69
	3. Do you eat three meals a day properly? Yes:1, No:0			0.89 ± 0.32	0.79 ± 0.41
	4. Did you find it harder to eat hard foods than it was six months ago? Yes:0, No:1			0.56 ± 0.50	0.75 ± 0.44
	5. Do you sometimes cough when drinking tea or soup? Yes:1, No:0			0.73 ± 0.45	0.75 ± 0.44
	6. Did you lose more than 2–3 kg in 6 months? Yes:1, No:0			0.82 ± 0.39	0.96 ± 0.20
	7. Do you think you walk slower than before? Yes:1, No:0			0.44 ± 0.50	0.54 ± 0.51
	8. Have you ever fallen in the last year? Yes:1, No:0			0.80 ± 0.41	0.70 ± 0.47
	9. Do you do exercise such as walking at least once a week? Yes:1, No:0			0.67 ± 0.47	0.57 ± 0.51
	10. Are you told by people around you that you have something to forget, such as “always hear the same thing”?			0.91 ± 0.29	0.79 ± 0.41
			Yes:0, No:1		
	11. Do you sometimes forget what month or day it is today? Yes:0, No:1			0.74 ± 0.44	0.58 ± 0.50
			Smoking	8.70%	16.70%
	12. Do you smoke?		Not smoking	63.00%	58.30%
			Stopped	28.30%	25.00%
	13. Do you go out at least once a week? Yes:1, No:0			0.89 ± 0.32	0.92 ± 0.28
	14. Do you interact with family or friends on a daily basis? Yes:1, No:0			0.89 ± 0.31	0.88 ± 0.34
	15. Do you have someone close to you when you feel sick? Yes:1, No:0			0.91 ± 0.28	0.88 ± 0.34

### Percent change in muscle mass

**Fig. 4:**
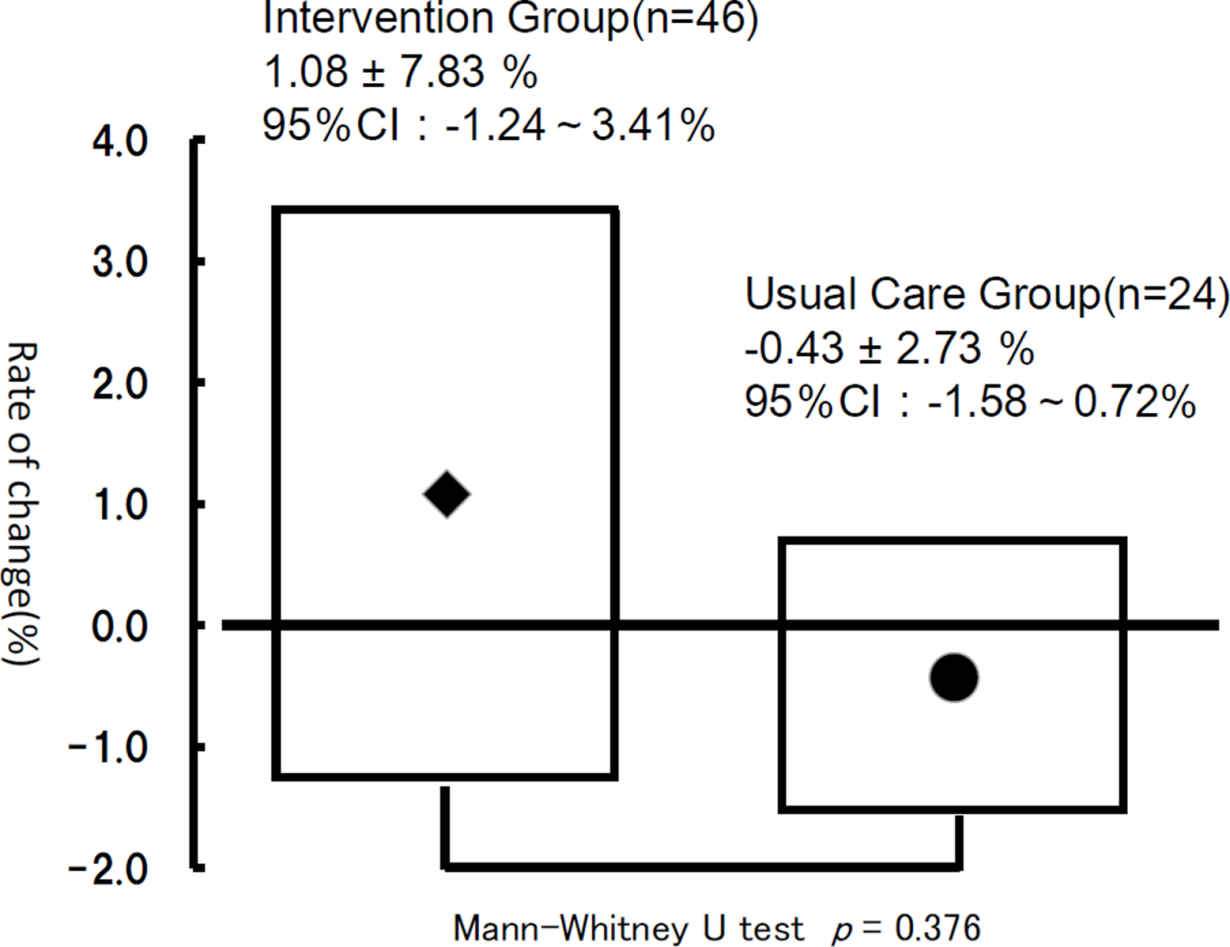
Rate of change in muscle mass between the intervention group and the usual care group

When we examined the percent (%) change in muscle mass at + 6 M as compared to the start point (enrollment), we found that IG was 1.08 ± 7.83 (95%CI: -1.24-3.41) and UG was − 0.43 ± 2.73 (95%CI: -1.58-0.72), indicating that, although there was a trend toward increased muscle mass as a result of pharmacist intervention, it was not statistically significant (p = 0.376; Fig. 4).

### Percent change in five Times Sit-To-Stand test time

When we examined the percent (%) change in the Five Times Sit-To-Stand Test times at + 6 M as compared to the start point (enrollment), we found that IG was − 0.02 ± 0.24 (95%CI: -0.09-0.05) and UG was − 0.04 ± 0.21 (95%CI: -0.13-0.07), indicating that there was no significant difference. However, in cases of patients whose second measured time was faster than their first, IG was 65.2% and UG was 29.2%, indicating a significant difference between the two groups (p = 0.00563; Table 3).

**Table 3:** Rate of change in secondary endpoints

Second time	Intervention	Usual Care	
	Group	Group	
Faster	30 (65.2%)	7 (29.2%)	
No change, slower	16 (34.8%)	17 (70.8%)	
Total	46 (100.0%)	24 (100.0%)	
Odds ratio: 4.48 *p* = 0.00563			

### Percent change in secondary endpoint items

When we examined the percent (%) change in the values measured at + 6 M as compared to the same values measured at enrollment (body weight, body fat percentage, BMI, estimated bone mass, body water percentage), we found that there were no major differences for any of the items (Table 4).

**Table 4:** Rate of change in secondary endpoints

Secondary evaluation items	sex	Group	n	Mean	95% CI	
Body weight	M	IG	25	-1.07%	-0.54% ～ -1.59%	
		UG	13	-1.93%	-1.16% ～ -2.70%	
	F	IG	21	-1.15%	-0.75% ～ -1.55%	
		UG	11	-4.94%	-3.55% ～ -6.32%	
Body fat percentage	M	IG	25	-0.07%	-0.09% ～ -0.05%	
		UG	13	-0.07%	-0.04% ～ -0.09%	
	F	IG	21	-0.02%	-0.03% ～ -0.01%	
		UG	11	-0.08%	-0.05% ～ -0.10%	
BMI	M	IG	25	-0.02%	-0.03% ～ -0.03%	
		UG	13	-0.02%	-0.02% ～ -0.03%	
	F	IG	21	0.00%	-0.01% ～ -0.001%	
		UG	11	-0.04%	-0.03% ～ -0.06%	
Estimated bone mass	M	IG	25	-0.01%	-0.02% ～ 0.00%	
		UG	13	0.00%	-0.01% ～ 0.01%	
	F	IG	21	0.00%	-0.01% ～ 0.01%	
		UG	11	-0.02%	-0.03% ～ -0.01%	
Body water content	M	IG	25	0.04%	0.03% ～ 0.05%	
		UG	13	0.02%	0.01% ～ 0.04%	
	F	IG	21	0.02%	0.01% ～ 0.04%	
		UG	11	0.05%	0.03% ～ 0.07%	

IG : Intervention Group UG : Usual Care Group

## Discussion

In this study, community pharmacies were two groups. The pharmacies were assigned randomly to either the intervention during medication guidance group (IG) or the usual medication guidance group (UG: control group). The subjects were older persons with chronic conditions who used the pharmacies in the two groups every month, and who were encouraged by the pharmacists to exercise at home when they were provided with their medication. We investigated the changes in their muscle mass and frailty improvement index six months after the start of the study (+ 6 M). The IG showed a trend toward increased muscle mass, which was the primary endpoint. The IG showed a significantly higher value in cases in which the time for the Five Times Sit-To-Stand test, which was one of the secondary endpoints, was faster the second time than the first time.

Eduardo et al. reported that strength training significantly increased muscle mass in people in their 90s who were at risk of falling and, as a result, reduced their incidence of falls [19]. This result suggests that increasing muscle mass may prevent frailty and sarcopenia and that the intervention by the pharmacists contributed to maintained patient muscle mass, preventing frailty.

We believe the reasons why we did not observe a statistically significant difference for the primary endpoint in this study were as follows: The first is the fact that there was variation in the way that the patients understood the information provided by the pharmacists; the second is the possibility that the period during which the efficacy of the intervention was determined (six months) may have been too short; the third is the fact that there were limitations to the detection ability that was assumed at the outset of the study. Concerning the first issue, although it has been reported that the muscle mass of those in elderly care facilities who undergo muscle strength training under the guidance of an expert trainer increases [19], our study respected the autonomy of the patients and we believe that this is what inevitably led to the wide variation in the results. Regarding the period used to determine efficacy, a study conducted in Taiwan reported improvements in frailty as a result of three months of exercise and nutritional consultation intervention [20]. However, even though it was a short period, it represents evidence that was obtained through careful intervention provided by an expert in a controlled environment. Thus, when using a method such as that used in the present study in which the patients’ autonomy is respected, we believe it is necessary to utilize more than six months to ensure that clearer results are obtained. However, during the six-month intervention period, the pharmacists encouraged the patients to exercise at home only six times, which we believe is a too short time to confirm whether or not there was the maintenance of or an increasing trend in muscle mass.

Indeed, it is known that muscle mass decreases as one ages, and it has been reported that among those in their eighties, total muscle mass decreases by 40–50% of that of those in their twenties when measured using the creatinine method [21]. The same trend is observed among Japanese people as well. It has been indicated that muscle mass is useful as an index that reflects the health status of elderly people [22]. If muscle mass is maintained through the widespread implementation of this kind of approach, it may not only maintain patient activity of daily living levels and reduce their risk of falls and fractures, which seriously decrease quality of life.

Another achievement of this study was that it demonstrated that pharmacists can provide guidance to patients in a limited number of pharmacies, within the time they have to provide it, in order to encourage them to take action. In other words, the pharmacists were able to utilize the nudge theory to motivate the patients to exercise on their own at home.

Nudge theory is also applied to health guidance, and a systematic review reported that it is possible to use nudge theory to change the health behaviors of diabetes patients [23]. In Japan, it has been reported that the use of the nudge theory by pharmacists in their dealings with diabetes patients is an effective form of lifestyle intervention [24]. Another study investigated the effectiveness of medication guidance provided to hypertension patients at community pharmacies that used nudge theory [25]. However, this is the first study to attempt to utilize the nudge theory at community pharmacies to encourage patients to exercise. We believe this study is of major significance because it found that simply by talking to pharmacists, and in the absence of an environment in which physical therapists and other specialists were able to control the conditions–— they were able to encourage patients to exercise on their own at home.

Although this was a pragmatic randomized controlled trial, it has the limitations of one intervention group and participants being randomized but not blinded.

The Japan Agency for Gerontological Evaluation Study (JAGES), a longitudinal study conducted in 2013—2016 reported that elderly individuals aged 75 years and older who participated in the “Kayoi-no-Ba” (“place to go”) program kept worsening their risk of requiring nursing care by 46% as compared to those who did not participate [26]. In Japan, there has been a tendency for patients to extend the period of time between hospital outpatient visits as a result of the COVID-19 pandemic, but we showed in the present study that by using an approach in which pharmacists engage with older persons with chronic conditions during the patients’ monthly visits to community pharmacies, it was possible to prevent decreases in muscle mass. This supports how important it is for elderly people to utilize the services of medical facilities and community pharmacies every month, as this enables them to maintain their links to society, and it indicates that community pharmacies can be utilized as “places to go” for elderly individuals.

Pharmacists are expected to play a role in a large number of fields, including filling prescriptions, the sale of over-the-counter (OTC) medications, and community health promotion. Another of these roles is in the prevention of frailty among older persons with chronic conditions. It is of major significance that pharmacists endeavor to prevent frailty through their routine pharmacy activities to allow older persons to undergo medical treatment and recovery from illness in a comfortable environment in the communities that they are used to. This study provides evidence for this.

## Conclusion

As super aging societies increasingly become a global problem, the prevention of frailty among the elderly is a major issue for those involved in medical care and nursing care. In this study, we were able to show that the provision of information to older persons with chronic conditions by community pharmacists about simple exercises that can be done at home during routine medication guidance and through the use of nudge theory leads to behavioral changes on the part of the patients.

## Data Availability

The data that supports the results of this study will be provided by the corresponding authors upon receipt of reasonable requests. Due to ethical restrictions and privacy concerns, each item of data will not be made available publicly.
